# Experience in the Management of Patients with Chronic Vascular Ulcers of the Lower Limbs Using Negatively Charged Polystyrene Microspheres

**DOI:** 10.1155/2020/3673657

**Published:** 2020-01-22

**Authors:** María Teresa Cacua Sánchez

**Affiliations:** Peripheral Vascular Surgeon Universidad El Bosque, Specialist in Management and Health Services, Universidad Sergio Arboleda, Bogotá, Colombia

## Abstract

**Background:**

Chronic ulcers of the lower limbs are a socioeconomic health problem, having a high incidence in the adult population. Despite a correct etiological treatment, in addition to the multiple lesion management options available, healing percentage and speed remain low, which makes it a great therapeutic challenge.

**Objective:**

To describe the outcome and effectiveness of the use of PolyHeal® Micro in the granulation and epithelialization of chronic ulcers of the lower limbs.

**Methods:**

Descriptive observational case series of 19 patients with diagnosis of chronic vascular ulcers of the lower limbs, treated at Medical Center Nuestra IPS and Medical Center Juan Pablo II in Bogotá between March 2018 and December 2018, who received PolyHeal® Micro as topical treatment for their lesions. Patients were assessed taking into account age, sex, ulcer size, pain, wound exudate, as well as granulation and epithelialization response.

**Results:**

In this series, the mean age of patients was 67.9 years, they were mostly females (84%), and the most common location of ulcers was the internal malleolus (50%). In terms of wound severity, 47% were found to be severe, 43% moderate, and 10% mild. The median time of lesion onset was 28 weeks, with a maximum of 2080 and a minimum of 8 weeks. In total, 87% of the ulcers were of venous etiology. After 12 weeks of treatment with Polyheal, ulcers showed a significative improvement of Wollina score means: 0.80 ± 0.90–5.90 ± 1.47; (*p* < 0.000). Wound area mean at the start of treatment was 31.6 cm^2^, and at the end of treatment was 17.85 cm^2^, which is equivalent to a wound area surface reduction of 54.2%, with a statistically significant *p* value. The interquartile range showed a reduction of 64.3% in the central means of wounds. Seventy percent of the patients reached a granulation percentage greater than 70%, 17% of the lesions exhibited an improvement of 30–70%, and there was an overall granulation improvement in 87% of the patients. Concerning epithelialization, 40% of patients reached a percentage higher than 70%, and 17% of patients between 30% and 70%. Treatment time was 12 weeks in 68% of cases, with an average response time of 8.1 weeks. Based on the visual analogue scale (VAS), a reduction in patients' perception of pain was achieved, dropping from an average of 6 (moderate to severe pain) to 2 (little pain), demonstrating an improvement in this regard. Fifty percent of the ulcers showed decreased exudate, resulting in a dry state. The patient satisfaction rate at the end of treatment was 89%.

**Conclusion:**

The use of PolyHeal® Micro for an average of 8.1 weeks of treatment showed a high rate of granulation and epithelialization in chronic ulcers of the lower limbs, improving pain perception in these patients and generating a high degree of treatment satisfaction.

## 1. Introduction

The skin is considered the largest organ in most vertebrates, having a complex structure made up of three layers: the epidermis, the dermis, and the hypodermis, which under normal conditions have the ability to regenerate themselves [1]. However, its integrity may be affected by multiple causes, including chronic wounds, which may be extensive and deep, altering the biological healing process and modifying the complex series of interactions existing between the different types of cells, mediating molecules, and components of the extracellular matrix that allow the growth and adequate regeneration of tissues [2].

Chronic ulcers affect 1% of the adult population and 3.6% of people over 65, rising to more than 5% in people over 80 [3].

These ulcers are due to different diseases; the most common ones are: chronic venous insufficiency constituting around 70% of leg ulcer presentations; chronic peripheral artery disease corresponding to 20%; and diabetes mellitus which accounts for 85% of foot ulcers leading to peripheral neuropathy, often accompanied with arterial disease [4].

In the course of a lifetime, almost 10% of the population will develop a chronic wound, with a wound-related mortality rate of 2.5% [5], and although wound healing is a natural process of regeneration, the speed of healing in most cases of a chronic wound is slow, as 20% will not heal in less than 2 years [6], and approximately 8% will not heal after 5 years, reaching a health cost of 7 billion dollars in the USA annually [7]. It is therefore a significant cause of morbidity worldwide, causing prolonged hospitalizations, diminishing quality of life, resulting in incapacity to work, and representing great economic and social costs.

Professionals such as general practitioners, dermatologists, vascular surgeons, geriatricians, nurses, and sometimes psychiatrists deal with this issue on a daily basis [8].

Although currently there are a variety of films, topical medications, dressings and gels that help to close these wounds, technological advances have focused on understanding the biomolecular process of the wound and creating appropriate products for mediating the correct stage of the repair process that is disrupted, thereby generating excellent conditions for the skin to resume the process of healing and self-regeneration.

Based on the above, PolyHeal® Micro has been developed, a substance composed of a suspension of negatively charged polystyrene microspheres (CPM) at a concentration of 4.5 × 10^6^ microspheres/mL, suspended in glycerol and phosphate buffer in water for topical use. This makes it a quick and easy-to-apply product, which may be readily adapted to any area of the ulcer and whose mechanism of action generates cell adhesion, thus promoting processes of granulation and epithelialization in chronic wounds, and achieving in a short period, according to studies, a rapid response in the evolution of wounds [9]. In this way, after closure, comprehensive management of the patient's underlying pathology may be continued.

## 2. Objective

To describe the outcome and effectiveness of the use of PolyHeal® Micro in the granulation and epithelialization of chronic ulcers of the lower limbs.

## 3. Materials and Methods

Descriptive observational case series.

### 3.1. Selection of Study Population

Patients were selected for the study based on the following criteria:

1. Patients with chronic vascular ulcers (over 4 weeks) of the lower limbs of venous, arterial, mixed, or diabetic etiology.
2. All patients with the above criteria who were admitted for vascular surgery at Medical Center Nuestra IPS and at Medical Center Juan Pablo II in Bogotá between March 2018 and December 2018, and who attended the consultation.

Exclusion criteria:

1. Patients with less than 4 weeks of evolution from onset of ulceration.
2. Patients with chronic ulcers of the lower limbs with signs of infection.

To assess lesion severity, the longest side of the wound was taken as a parameter and classified according to Table 1.

### 3.2. PolyHeal® Micro Application Protocol

The number of bottles of PolyHeal® Micro 7.5 ml to be applied for each patient was determined based on the size of the ulcer, as defined by the outpatient specialist.

All patients received oral and written information about the treatment to be performed, and an informed consent was signed. The age, sex, anatomical location of the ulcers (internal malleolus, external malleolus, anterior tibia, and dorsum of the foot), time of lesion evolution, TIME model (tissue, infection/inflammation, moisture, and edge), wound pain according to the visual analogue scale, and size in terms of length and width (cm) were recorded. These measures were taken at the start of, during, and after treatment.

All patients underwent the same application process, which was explained by the treating specialist both to the patient and to the caregiver in case there was one. They received in writing the step-by-step instructions for the healing of the wound, and follow-up was performed by the specialist every 8 days, where pictures were taken following this application protocol:

Specialist protocol (at the beginning of treatment and every 8 days)

- Clean the wound with 500 cc of normal saline.
- Dry the wound with sterile gauze.
- Measure the lesion: length, width and depth in cephalocaudal direction.
- Evaluate lesion tissues according to TIME (viable tissue, signs of infection and inflammation, exudate, and perilesional edges).
- Take a photographic record.
- Shake the PolyHeal® Micro bottle.
- Apply PolyHeal® Micro to the entire lesion.
- Cover lesion with gauze impregnated with PolyHeal® Micro.
- No other type of product is applied around the lesion or its edges.
- Use dry gauze as a second dressing.
- Position cotton bandages and elastic bandage are positioned and compression is applied based on the presence and verification of pedal pulses, capillary filling and distal perfusion, fix them with adhesives.
- Give recommendations to patient and family member or caregiver on home care, and strengthen education in each session on nutrition, hygiene, administration of medications prescribed by the physician, physical activity, rest, leisure time, sexuality, bandage care at home, and warning signs.
- Check comfort of the bandage and general condition of the patient before release.
- Explain to the patient that he or she should clean the wound once a day every day of the week and that the bottle should be used in its entirety and discarded once the entire product has been applied.
- Schedule appointment within 8 days.

Patient and/or caregiver protocol for daily treatment

- Clean the wound with 500 cc of normal saline.
- Dry the wound with sterile gauze.
- Shake the PolyHeal® Micro bottle.
- Apply PolyHeal® Micro to the entire lesion.
- Cover lesion with gauze impregnated with PolyHeal® Micro.
- No other type of product is applied around the lesion or its edges.
- Use dry gauze as a second dressing.
- Position cotton bandages and elastic bandage are positioned and compression is applied based on the presence and verification of pedal pulses, capillary filling and distal perfusion, fix them with adhesives.
- Discard the empty bottle of PolyHeal® Micro.
- This process is carried out once a day, every day of the week.

The frequency of application was established at once a day, up to the total use of the formulated bottles.

## 4. Statistical Analysis

### 4.1. Demographic Characterization

The study population consisted of 19 patients with 30 vascular lesions in the lower limbs.

**Figure 1:**
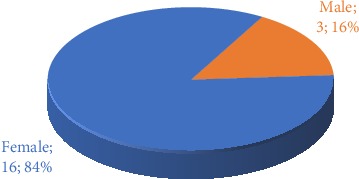
Population distribution by sex.

This population exhibited the following demographic characteristics:

The predominant sex of the study population is female, with more than 80%, as shown in Figure 1 and Table 2.

The average age in the study population was 67.9 years, with a maximum age of 90 and a minimum of 32. The female group included the oldest individuals, with an average age of 68.5 years, compared to 63.7 years for men.

**Figure 2:**
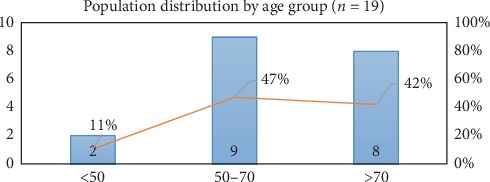
Population distribution by age group.

As may be observed in the following graph, the group of individuals over 50 years of age accounts for 89% of the population included in the study, and the group of 50–70 years constitutes the largest concentration of individuals.  Figure 2.

**Figure 3:**
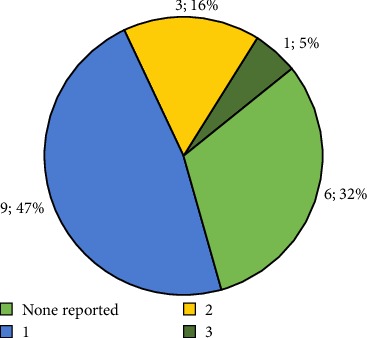
Number of associated pathologies per patient.

Based on population characteristics, chronic non-communicable pathologies were found in 68% of the population, with arterial hypertension being the most prevalent (Figure 3 and Table 3).

### 4.2. Lesion Characterization

Of the 30 lesions found in the 19 patients studied, the largest proportion corresponded to venous ulcers (Table 4).

**Figure 4:**
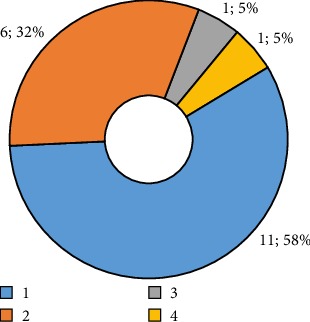
Number of lesions per patient.

In terms of the number of lesions per patient, it was found that more than 80% of the population had one or two lesions and 10% between 3 and 4 lesions (Figure 4).

### 4.3. Anatomical Location of Lesions

The ulcers were usually located anatomically on the lower and internal region of the lower limbs. In this study, these were mostly observed at the level of the internal and external malleoli with 70%, followed by the anterior tibia with 19% (Table 5).

**Figure 5:**
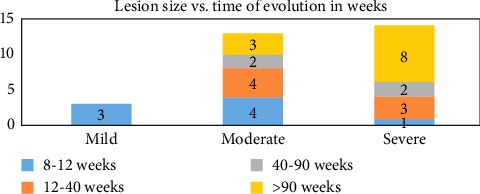
Time of evolution of lesions.

Regarding the time of onset of the vascular pathology in the study population, it was observed that 37% of the lesions were over 90 weeks old. The group of 8–40 weeks had the greatest number of lesions, accounting for 50% of the cases. In relation to lesion size, the largest lesions were concentrated in those with for more than 90 weeks, accounting for 26% of the cases (Table 6 and Figure 5).

## 5. Intervention Results

Sixty-eight percent of treated patients received 12 weeks of treatment with PolyHeal® Micro, whereas 16% received treatment for 9 weeks. The variation between the weeks of treatment was determined by the percentage of lesion granulation, since upon reaching expected granulation, the patient stopped receiving the product; the average time of appearance of results was 8.1 weeks (Table 7).

### 5.1. Weekly Assessment of the Wollina Score and Wound Area Surface

After 12 weeks of treatment with Polyheal (Table 8), ulcers showed a significative improvement of Wollina score means: 0.80 ± 0.90–5.90 ± 1.47; (*p* < 0.000). Wound area mean at the start of treatment was 31.6 cm^2^, and at the end of treatment was 17.85 cm^2^, which is equivalent to a wound area surface reduction of 54.2%, with a statistically significant *p* value. The interquartile range showed a reduction of 64.3% in the central means of wounds.

A Friedman's test was used to compare non-parametric variables and a Student's *t*-test was used to compare parametric variables.

Friedman's test *X*_2r_ 185.2

*H*_0_ *hypothesis*: Treatment does not improve ulcers' granulation.

*H*_1_ *hypothesis*: Treatment improves ulcers' granulation.

*P* = 0.001.

The alternative hypothesis is accepted: Polyheal improves ulcers' granulation.

The use of PolyHeal® Micro is expected to accelerate the production of granulation tissue and lesion epithelization through negatively charged polystyrene microspheres, which have an effect on the inflammatory phase and improve the proliferative phase of healing for tissue repair.

**Figure 6:**
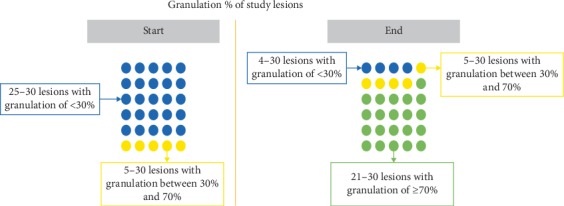
Results obtained with the use of PolyHeal**® **Micro: granulation.

**Figure 7:**
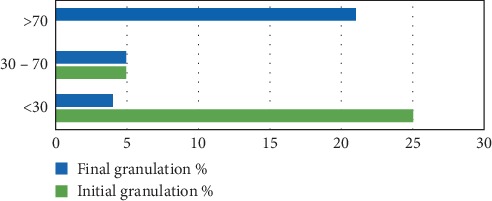
Comparison granulation % at start and end of treatment.

In the study population, following the use of PolyHeal® Micro, 70% of lesions reached a granulation level greater than 70% (Figures 6 and 7) and 17% of wounds achieved a granulation percentage between 30% and 70%; only 13% remained with a granulation percentage below 30%.

**Figure 8:**
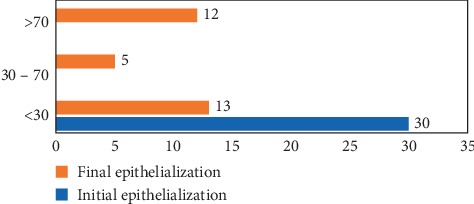
Comparison epithelialization % at start and end of treatment.

As for the epithelialization of lesions at the end of treatment with PolyHeal® Micro, 57% of the lesions in the study reached an epithelialization level between 30% and 70% (Figure 8).

When evaluating the final granulation and epithelialization results, the following was found (Table 9):

- Mild lesions reached 67% of granulation and epithelialization greater than 70%.
- Moderate lesions reached 69% of granulation, and 46% reached an epithelialization greater than 70%.
- Severe lesions reached 71% of granulation, and 29% reached an epithelialization greater than 70%.

## 6. Statistical Testing

### 6.1. For Lesion Epithelialization

Wilcoxon's statistical test was carried out with a 95% confidence interval, noting that due to the sample size of the statistic *T* where the data are adjusted to a normal distribution, the statistic *Z* is calculated to accept the hypotheses presented.

*H*_0_: No significant changes in wound size before and after the use of Polyheal

*H*_A_: Significant changes in wound size before and after the use of Polyheal

(1) $$\begin{array}{c} \begin{array}{c} H_{0}=M_{\text{ed}}=0,\\ H_{\text{A}}=M_{\text{ed}}\neq 0, \end{array} \end{array}$$

(2) $$\begin{array}{c} Z=\frac{t-N\left(N+1\right)/4}{\sqrt{N\left(N+1\right)\left(2N+1\right)/24}}=N\left(0.1\right)Z=-4.45. \end{array}$$

For the analysis of the study sample, the alternative hypothesis was accepted, which indicates that the positive results obtained in terms of wound size with the use of PolyHeal® Micro are statistically significant with *α* = 0.05.

**Figure 9:**
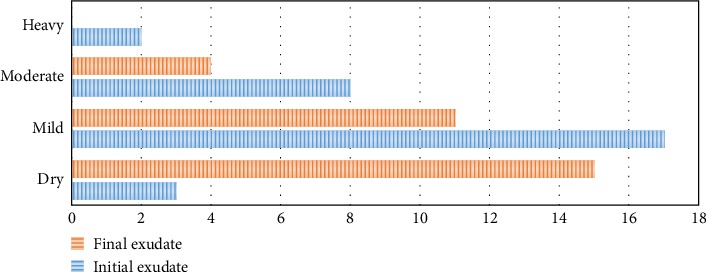
Comparison exudate at start and end of treatment with PolyHeal**® **Micro.

Concerning the lesion exudate, a significant improvement was noted since at the end of sn no heavy exudate was observed, and 50% of the wounds became dry (Figure 9).

**Figure 10:**
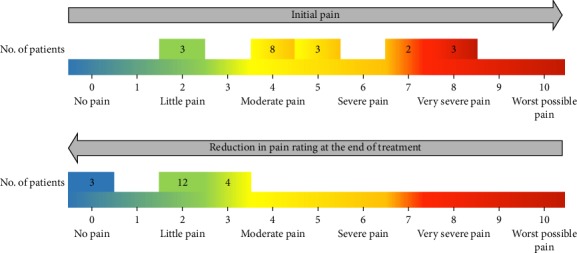
Comparison initial pain vs. final pain rating.

During the study, the visual analogue scale was applied to patients on admission and at the end of treatment. On admission, patients rated the level of lesion pain, reaching an average of 6 or severe pain. Once the intervention was concluded, the measurement was performed again, revealing a reduction in pain in all patients who, on average, reached a score of two. This corresponds to little pain, which proves to be a significant change in this regard (Figure 10).

## 7. Statistical Testing

### 7.1. To Measure Pain

Wilcoxon's statistical test was carried out with a 95% confidence interval, noting that due to the sample size of the statistic *T* where the data are adjusted to a normal distribution, the statistic *Z* is calculated to accept the hypotheses presented.

*H*_0_: No significant changes in wound pain severity before and after the use of PolyHeal® Micro.

*H*_A_: Significant changes in wound pain severity before and after the use of PolyHeal® Micro.

(3) $$\begin{array}{c} \begin{array}{c} H_{0}=M_{\text{ed}}=0,\\ H_{\text{A}}=M_{\text{ed}}\neq 0. \end{array} \end{array}$$

(4) $$\begin{array}{c} Z=\frac{t-N\left(N+1\right)/4}{\sqrt{N\left(N+1\right)\left(2N+1\right)/24}}=N\left(0,1\right)Z=-4.54. \end{array}$$

For the analysis of the study sample, the alternative hypothesis was accepted, which indicates that the results obtained in terms of pain reduction with the use of PolyHeal® Micro are statistically significant with *α* = 0.05.

According to the results obtained, 89% of the intervened patients showed an increase in the level of satisfaction, compared to previous treatments and to study admission (Table 10).

## 8. Discussion

Wound healing involves a complex physiological process aimed at restoring the integrity of the skin, as any impairment in its barrier function generates disturbance in the homeostasis and general well-being of the patient [11].

Chronic wounds continue to be a great medical challenge and, despite the technological advances and various treatments for their cure, they are still pathologies that generate great disability, morbidity and mortality, directly impacting health expenditure. They are a major public health problem because their treatment is expensive and may involve prolonged periods of hospitalization and additional surgical procedures.

Thus, chronic wounds constitute one of the greatest health care problems affecting the health system. They decrease the quality of life of those who develop them and their caregivers [12] and represent a high economic cost, based on wound treatment period, which according to Fernández [13], may vary between 150 and 180 days to achieve an effective recovery, coupled with the time spent by the nursing professionals responsible for the various treatments.

Based on the literature, if a wound does not respond to standard care (a response typically defined as 30% reduction in wound size for venous leg ulcers and 50% reduction for diabetic foot ulcers, within 4 weeks after treatment initiation), a change in treatment and use of specialized technology [14, 15] is indicated. Additionally, it should be borne in mind that the treatment of chronic ulcers has a double aspect: The underlying disease and local wound treatment [16], as the risk of such lesions recurring must always be minimized.

Finally, the constant technological development of different useful products for wound healing offered by the market, together with the progressive development of knowledge about molecular biology of wound healing, has demonstrated the importance of approaching this pathology with a treatment that not only reduces healing time and improves the patient's quality of life and morbidity, but also reduces costs. As such, PolyHeal® Micro is a substance that has an ability to replicate the functions of the extracellular matrix and promotes cell growth and proliferation, achieving a biochemical balance in the lesion. It increases the synthesis of collagen and the number of keratinocytes and endothelial cells, promoting the wound granulation process. It may be considered as a useful product in reducing healing time, with optimal results in improving the wound healing process.

## 9. Conclusion

The use of PolyHeal® Micro promotes the formation of granulation tissue, by increasing the percentage of wound healing. This therapy constitutes a major advance in the care of chronic wounds and has the potential to significantly improve the condition of most patients, without causing adverse events and showing good tolerability. The therapy is adjuvant to the management of the underlying pathology of the patient. It is an innovative and easy-to-use treatment that has demonstrated favorable results in terms of reduction of healing time, pain, and size of chronic wounds of the lower limbs, achieving a granulation percentage of more than 70% in 70% of patients, with a percentage of overall improvement in granulation of 87%.

Concerning epithelialization, 40% of patients reached a percentage higher than 70%, and 17% of patients between 30% and 70%. The overall percentage of epithelialization was 57%. Additionally, a significant decrease in pain was achieved in most patients, as well as a reduction in the production of exudate, thus obtaining a treatment satisfaction index of 89%.

## 10. Clinical Cases

RLL: Lower right limb.

LLL: Lower left limb.

Case 1.

**Figure 11:**
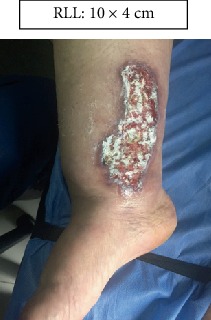
Start. Lower right limb.

**Figure 12:**
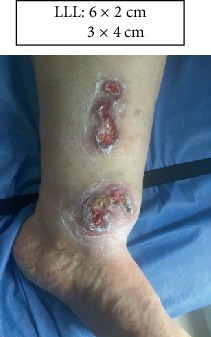
Start. Lower left limb.

Start (Figures 11 and 12).

**Figure 13:**
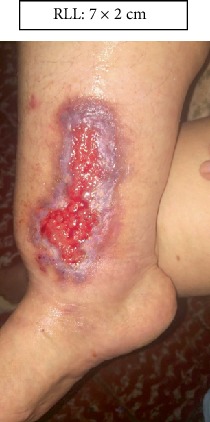
Week 4. Lower right limb.

**Figure 14:**
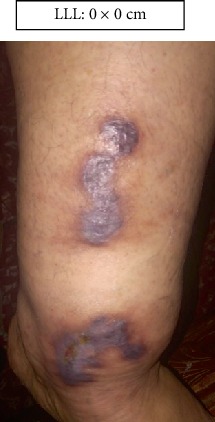
Week 4 . Lower left limb.

Week 4 (Figures 13 and 14).

**Figure 15:**
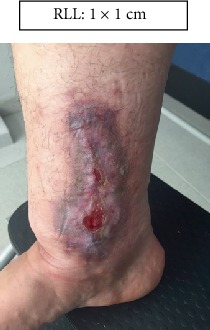
Week 9. Lower right limb.

**Figure 16:**
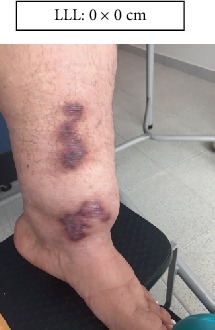
Week 9. Lower left limb.

Week 9 (Figures 15 and 16).

**Figure 17:**
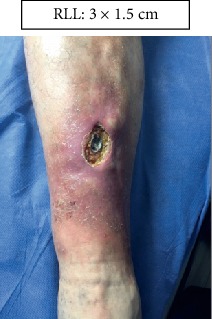
Start. Lower right limb.

**Figure 18:**
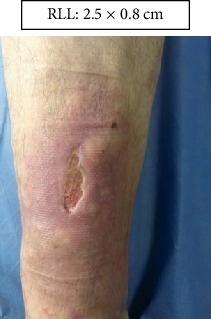
Week 5. Lower right limb.

**Figure 19:**
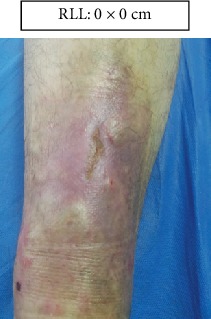
Week 10. Lower right limb.

Case 2 (Figures 17–19).

**Figure 20:**
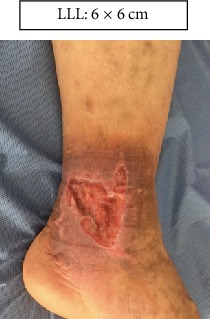
Start. Lower left limb.

**Figure 21:**
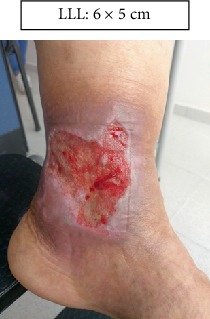
Week 6. Lower left limb.

**Figure 22:**
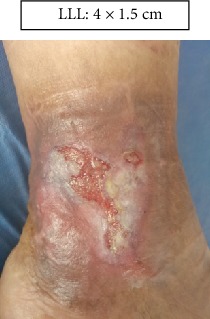
Week 12. Lower left limb.

Case 3 (Figures 20–22).

## Figures and Tables

**Table 1:** Ulcer classification based on diameter.

Ulcer classification based on diameter: [10]
(i) Mild: <2 cm
(ii) Moderate: 2–6 cm
(iii) Severe: >6 cm

**Table 2:** Distribution by sex.

Sex	No.	%
Female	16	84
Male	3	16

Total	19	100

**Table 3:** Associated pathologies.

Associated pathologies	
Vascular brain disease	1
Vascular brain disease + Hyperthyroidism	1
High blood pressure	8
High blood pressure + Heart disease	1
High blood pressure + Diabetes mellitus	1
High blood pressure + Diabetes mellitus + Obesity	1
None reported	6

Total	19

**Table 4:** Distribution by type of lesion.

Type of lesion	No.	%
Mixed ulcer	4	13
Venous ulcer	26	87

Total	30	100

**Table 5:** Anatomical location of lesions.

	No. lesions	%
Internal malleolus	15	50
External malleolus	6	20
Anterior tibia	4	13
Inner tibia and internal malleolus	2	6
Inner tibia	1	3
Inner tibia and external malleolus	1	3
Dorsum of the foot	1	3

Total	30	100

**Table 6:** Time of evolution of lesions.

Time of evolution of lesions in weeks	Lesion size			Total
	Mild	Moderate	Severe	
8–12	3	4	1	8
12–40		4	3	7
40–90		2	2	4
>90		3	8	11

Total	3	13	14	30

**Table 7:** Treatment time with PolyHeal® Micro.

Treatment time		
No. weeks	No. patients	%
3	1	5.0
7	1	5.0
9	3	16
10	1	5.0
12	13	68.0

Total	19	100

**Table 8:** Wollina score.

	Week 0		Week 4		Week 8		Week 12		*p* value^∗^
	Mean	SD	Mean	SD	Mean	SD	Mean	SD	
Granulation tissue (maximum 4)	0.3	0.47	1.93	0.94	2.67	0.96	3.27	0.87	0.000000
Color (maximum 2)	0.6	0.62	1.23	0.50	1.43	0.57	1.77	0.43	0.000000
Consistency (maximum 1)	0	0	0.1	0.31	0.6	0.50	0.87	0.35	0.000000
Total score wollina (maximum 7)	0.87	0.90	3.13	1.46	4.67	1.63	5.90	1.47	0.000000
Surface area (cm^2^)	31.36	31.82	25.58	28.77	20.52	27.25	17.85	26.56	0.000164

^∗^Statistically significant (*p* < 0.05).

**Table 9:** Final results by lesion size.

Lesion size at start of treatment	Final granulation			Final epithelialization		
	<30	30–70	>70	<30	30–70	>70
Mild (<2 cm)	1		2	1		2
Moderate (2–6 cm)	2	2	9	5	2	6
Severe (>6 cm)	1	3	10	7	3	4
Total	4	5	21	13	5	12

**Table 10:** Patient satisfaction results.

Patient satisfaction with treatment from study admission	
Increase	17
No variation	2

## Data Availability

The data used to support the findings of this study are available from the corresponding author upon request.
